# Supplementary material for: Stem cell proliferation is induced by apoptotic bodies from dying cells during epithelial tissue maintenance
Source: Nat Commun. 2019 Mar 5;10:1044. doi: 10.1038/s41467-019-09010-6 (PMC6400930; doi:10.1038/s41467-019-09010-6)
Supplement: Supplementary file 1 — Supplementary Information [file 41467_2019_9010_MOESM1_ESM.pdf]

Supplementary Information for:

Stem Cell Proliferation Is Induced By Apoptotic Bodies From Dying Cells During Epithelial Tissue Maintenance

CK Brock et al., *Nature Communications*

Supplementary Figure 1

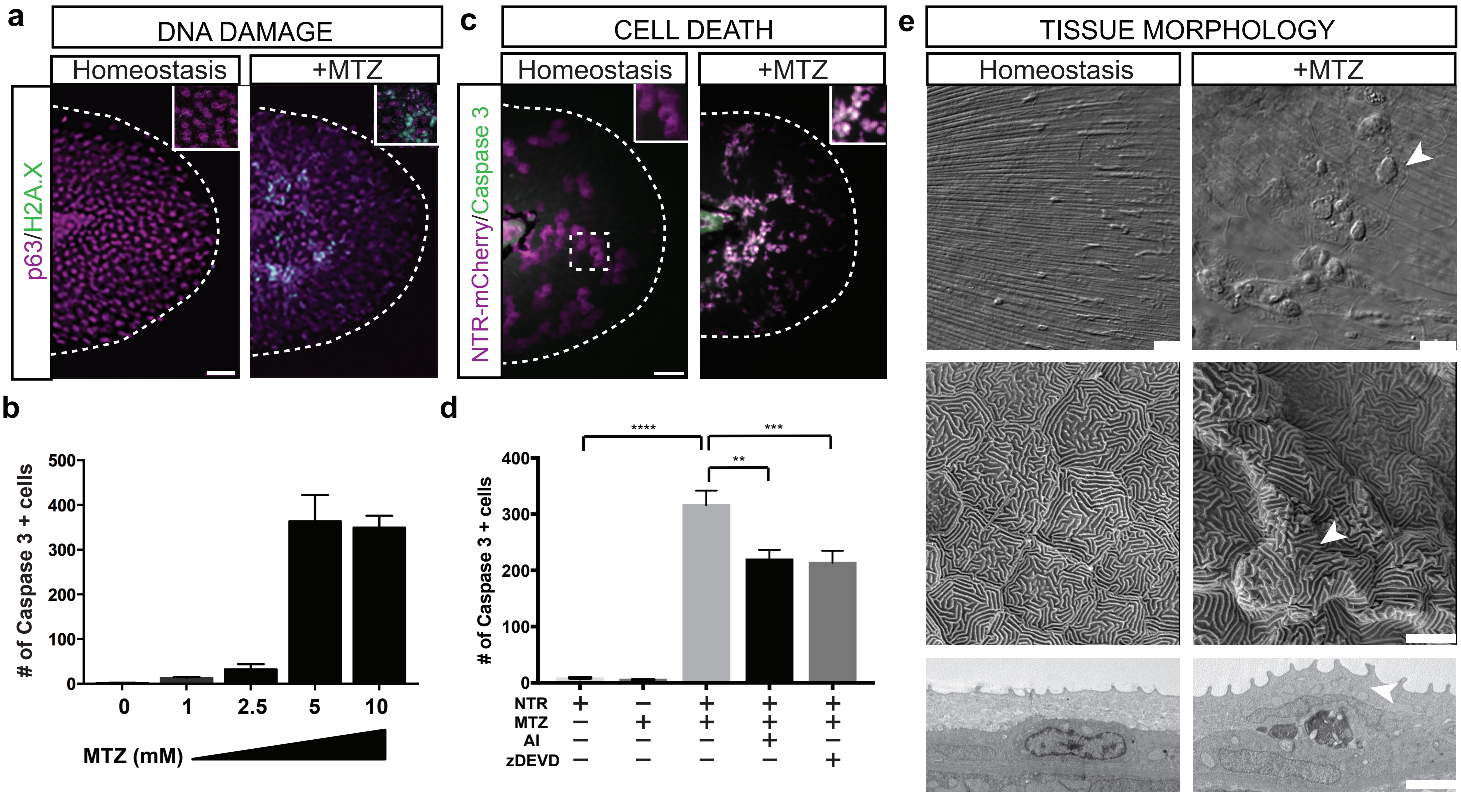

Supplementary Figure 1. DNA Damage and Apoptosis in p63-positive Epithelial Stem Cells. (a) Maximum intensity projection confocal images of zebrafish larvae immunostained for gamma H2A.X, a marker of DNA damage, and p63, that labels epithelial stem cells (scale = 100µm). (b) Quantification of activated caspase-3 positive cells in the tail fin epithelium after addition of increasing amounts of MTZ. (c) Fluorescent microscopy images reveal an increase in apoptosis after MTZ treatment (scale = 100µm) (d) Quantification of activated caspase-3 positive cells after treatment with MTZ and apoptosis inhibitor (AI) or caspase inhibitor (zDEVD). Mean number of positive cells from three independent experiments per time point/condition are plotted. Error bars represent s.e.m. **** $p<0.0001$, *** $p = 0.0007$, ** $p = 0.0027$. One-way ANOVA with Dunnett's mutiple comparisons test. (e) Brightfield microscopy shows changes in epithelial surface texture after MTZ treatment (scale = 50µm). Scanning electron microscopy (SEM) reveals that the surface periderm remains intact despite swollen basal epithelial stem cells during apoptosis (scale = 10µm). Transmission electron microscopy (TEM) of the epithelial bilayer shows that apoptotic basal cells push against the outer epithelium and reside between the basal layer and outer periderm (scale = 2µm).

Supplementary Figure 2

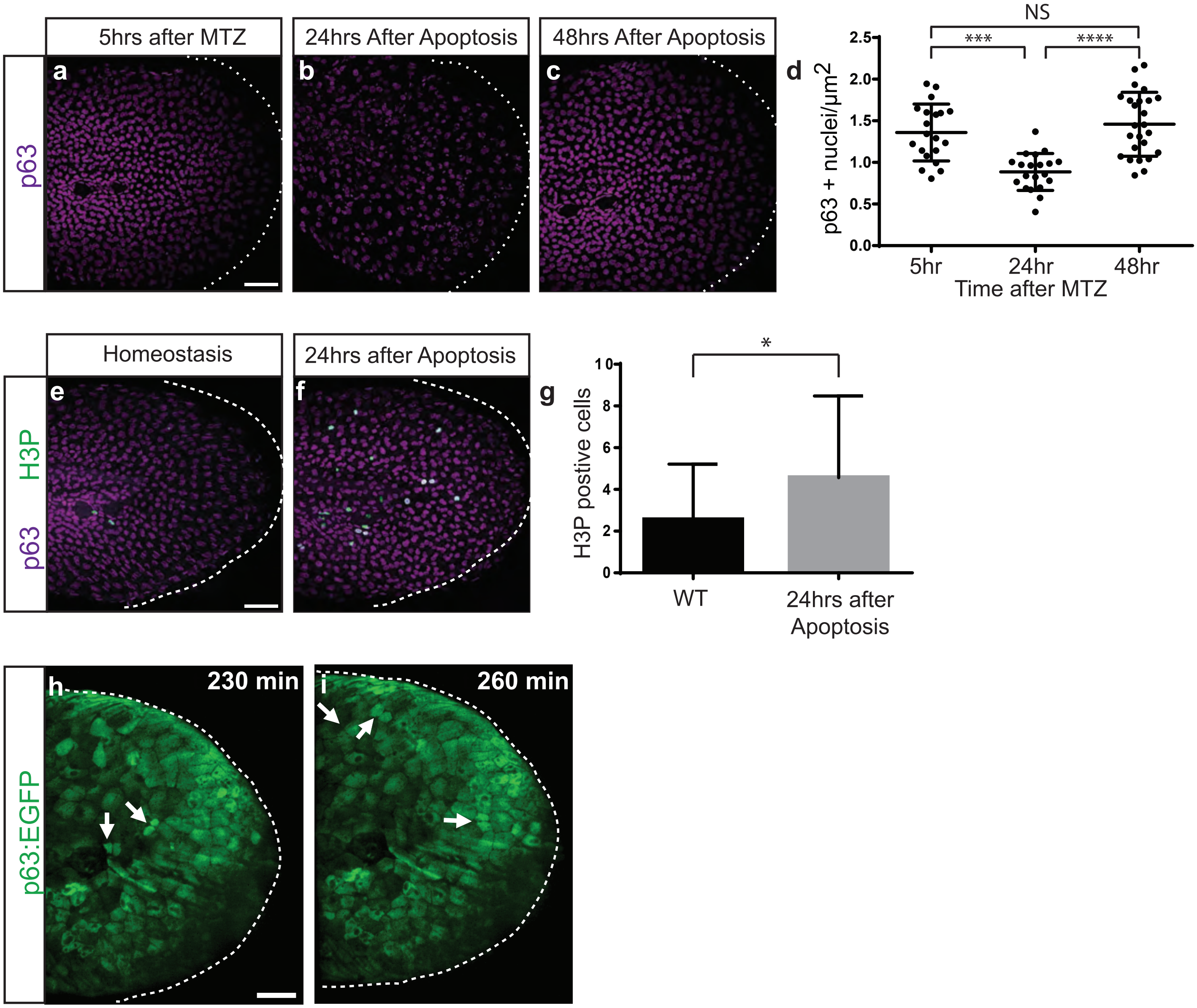

Supplementary Figure 2. Induced-Apoptosis Leads To Proliferation Of Surrounding p63-Positive Basal Stem Cells. (a-c) Maximum intensity projection confocal images of zebrafish tail fin epithelium at different times after MTZ treatment stained for the epithelial stem cell marker p63 (scale = 100µm). (d) Quantification of number of p63-positive stem cells per area (µm2). Mean number of p63-positive cells from three independent experiments per time point are plotted. Error bars represent SD. **** p <0.0001, *** p = 0.0002. One-way ANOVA with Tukey's mutiple comparisons test. (e-f) Maximum intensity projection confocal images of wild-type and MTZ-treated zebrafish larvae stained for phosphor-histone H3 (H3P) to mark mitotically active cells (scale = 100µm). (g) Quantification of mitotically active cells in the tail fin epithelium. Error bars represent SD. * p = 0.04. Unpaired two-tailed t-test. (h-i) Maximum intensity projection images from timelapse confocal microscopy of p63:EGFP-positive epithelial stem cells after MTZ treatment and washout (scale = 50µm).

Supplementary Figure 3

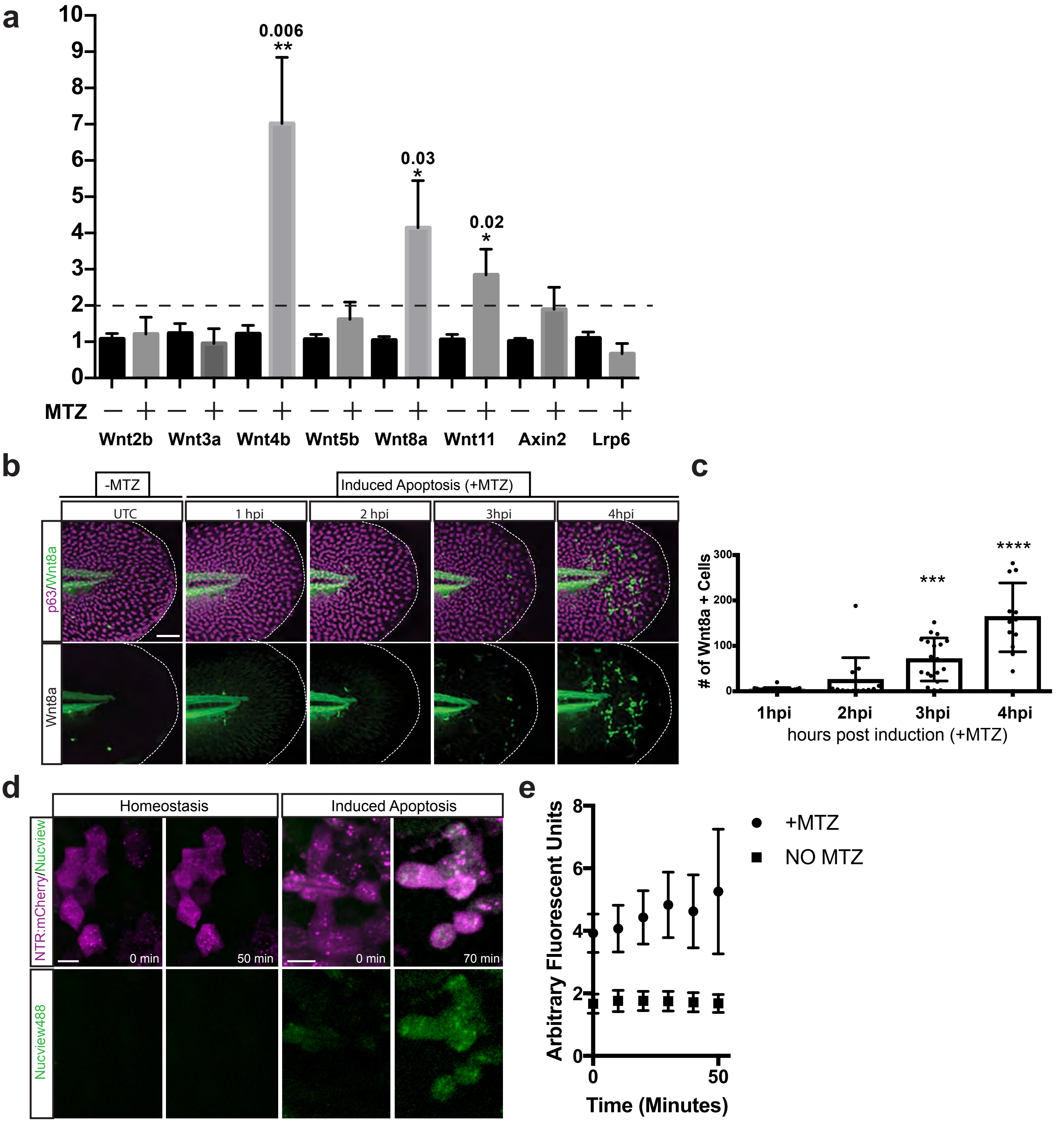

Supplementary Figure 3. Apoptosis-Induced Wnt signals and Production of Apoptotic Bodies. (a) Quantitation of mRNA levels for Wnt signaling pathway members (Wnt2b, Wnt3a, Wnt4b, Wnt5b, Wnt8a, Wnt11, Axin2, and Lrp6) at 5hr after addition of MTZ, normalized to β-actin. Data from three independent biological replicates. Error bars represent SD. Unpaired two-tailed t-test. (b) Maximum intensity projections of Wnt8a (green) and p63 (magenta) after induction of apoptosis (scale = 50µm). (c) Quantification of Wnt8a positive cells at different time points after induction of apoptosis. Mean number of Wnt8a positive cells from three independent experiments per time point are plotted. ***$p=0.0003$, ****$p<0.0001$. One-way ANOVA with Tukey's mutiple comparisons test. (d) Maximum intensity projections from a timelapase movie of the activate caspase 3 indicator Nucview (green) before and after MTZ treatment. (e) Quantification of Nucview fluorescent intensity within mCherry positive cells over time. Data from three independent movies. Error bars represent SD.

Supplementary Figure 4

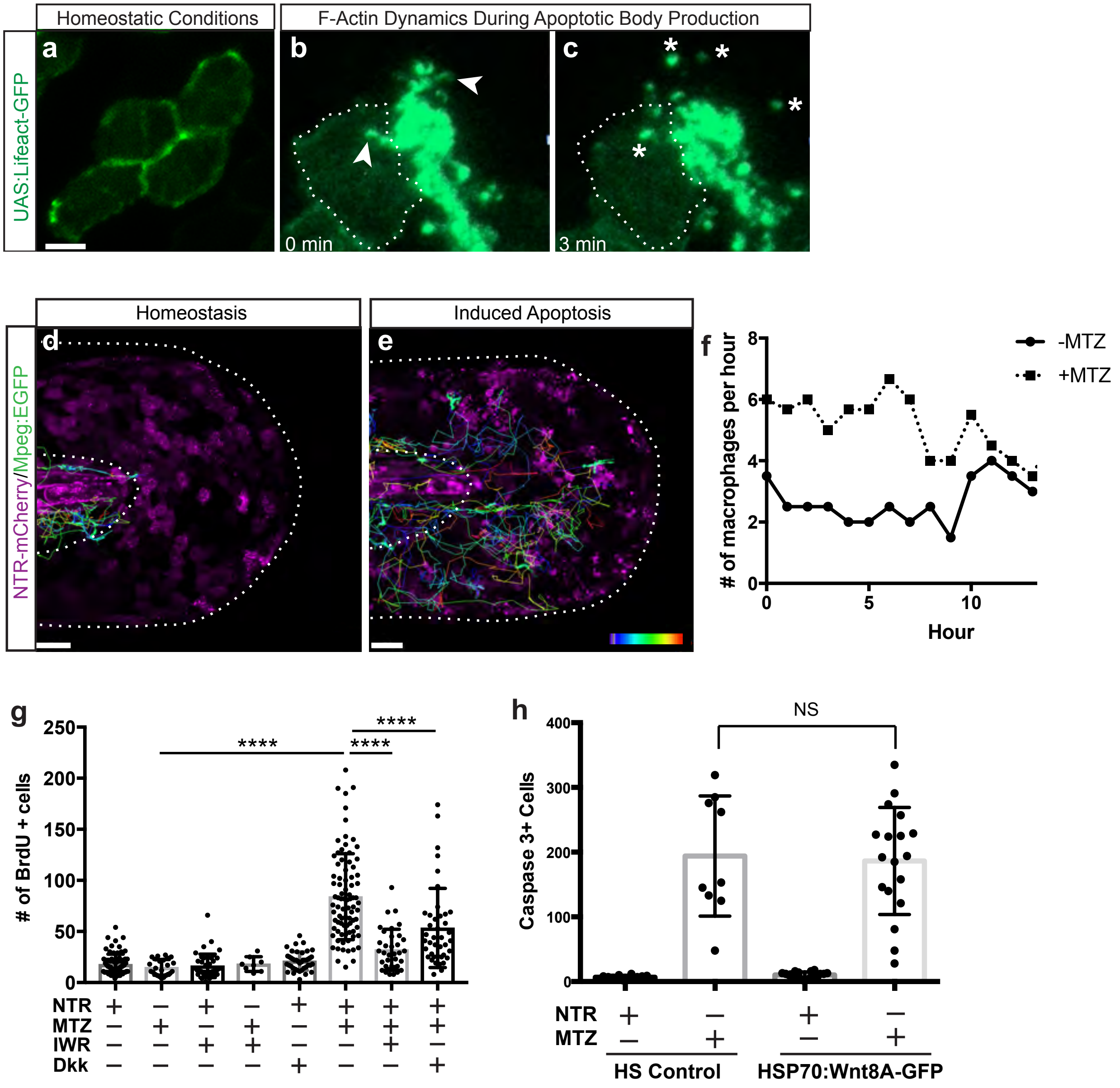

Supplemental Figure 4. Apoptotic Body Clearance and Modulation of Wnt Signaling. (a-c) Maximum intensity projections from a timelapse movie of *Et(Gal4-VP16)^{zc1036a};Tg(UAS-E1b:Lifeact-EGFP)* before and after MTZ treatment (scale = 10µm). (b) Filopodia (arrowheads) with an apoptotic body attached extends into a neighboring cell. (c) The apoptotic body (asterisk) is retained in the neighboring cell after retraction of the actin-based filopodia. Multiple apoptotic bodies (asterisks) are generated over the course of 3 minutes. (d-e) Maximum intensity projections from a timelapse movie of *Et(Gal4-VP16)^{zc1036a};Tg(mpeg:EGFP)^{gl22}* under homeostatic conditions or after induced apoptosis (scale = 40µm). Paths and displacment vectors are shown for individual cells. Color denotes time. (f) Quantification of mpeg positive macrophages in the epidermis (outside of area denoted by dotted line) each hour after the induction of apoptosis. Each time point represents an average from three independent movies. (g) Quantification of BrdU positive cells after induced apoptosis combined with overexpression of the Wnt inhibitor, Dkk1, or the chemical inhibitor of Wnt signaling, IWR. Error bars represent SD. **** p<0.0001. One-way ANOVA with Tukey's mutiple comparisons test. (h) Quantification of caspase-3 positive cells after heat-shock to induce Wnt8a-GFP expression shows cell death is not changed.

Supplementary Figure 5

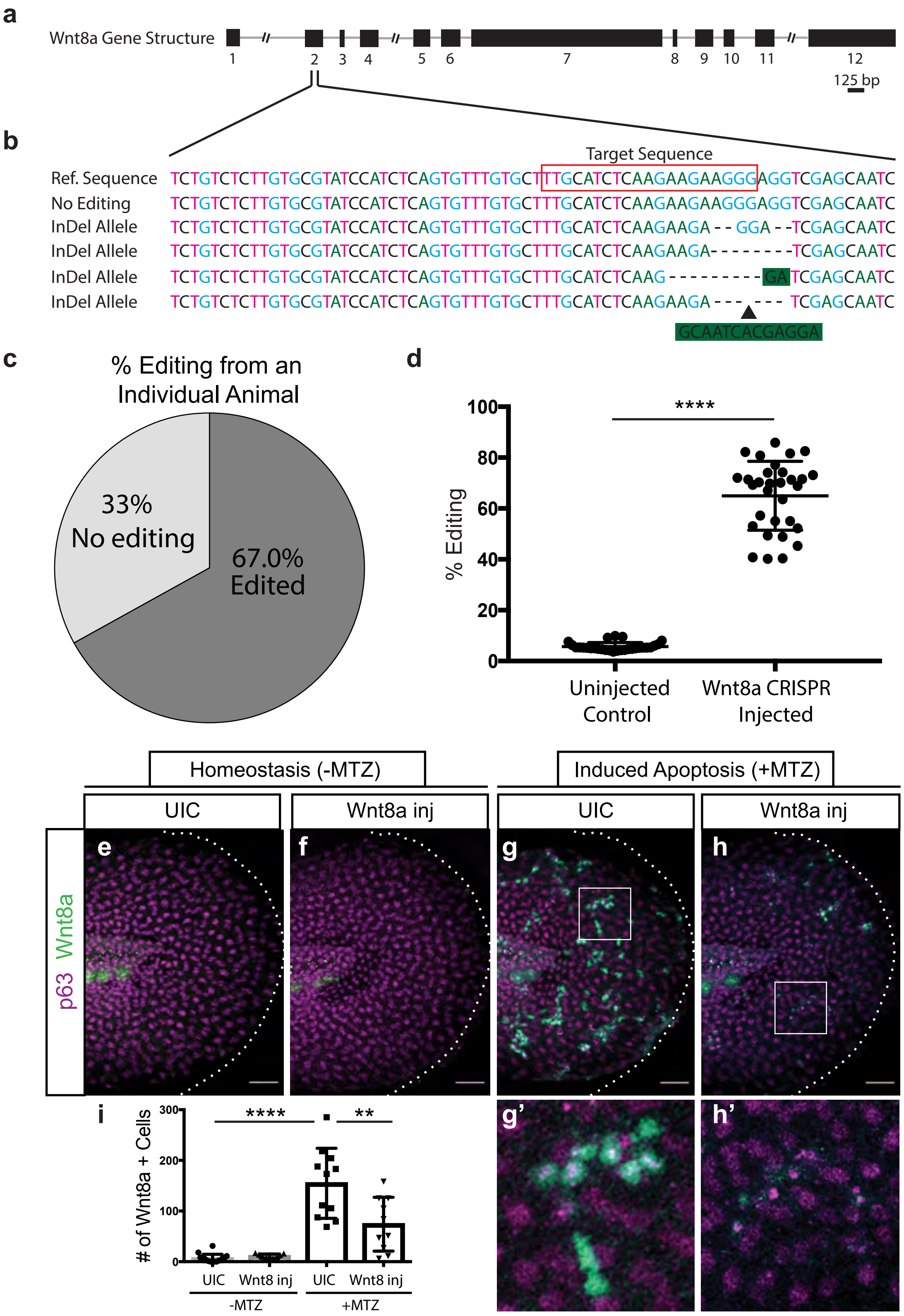

Supplementary Figure 5. Genetic Perturbation of Wnt8a During Homeostasis and After Induced Apoptosis. (a) Gene structure of Wnt8a. (b) Representative insertions and deletions observed by deep sequencing in exon 2 of Wnt8a. (c) The percentage of edited Wnt8a alleles from a representative individual animal. (d) The mean percentage of edited Wnt8a alleles from multiple individual larvae (n=29 UIC, n=31 Wnt8a inj). Error bars represent SD. ****p<0.0001. Unpaired two-tailed t-test. (e-h) Immunostaining for Wnt8a in uninjected (UIC) and Wnt8a CRISPR injected larvae during homeostasis and after induced apoptosis (scale = 50µm). (i) Quantification of Wnt8a positive cells during homeostasis and after induced apoptosis. Mean number of Wnt8a positive cells from three independent experiments per time point are plotted. ***p=0.0004, ****p<0.0001. Error bars represent SD. One-way ANOVA with Tukey's mutiple comparisons test.

Supplementary Figure 6

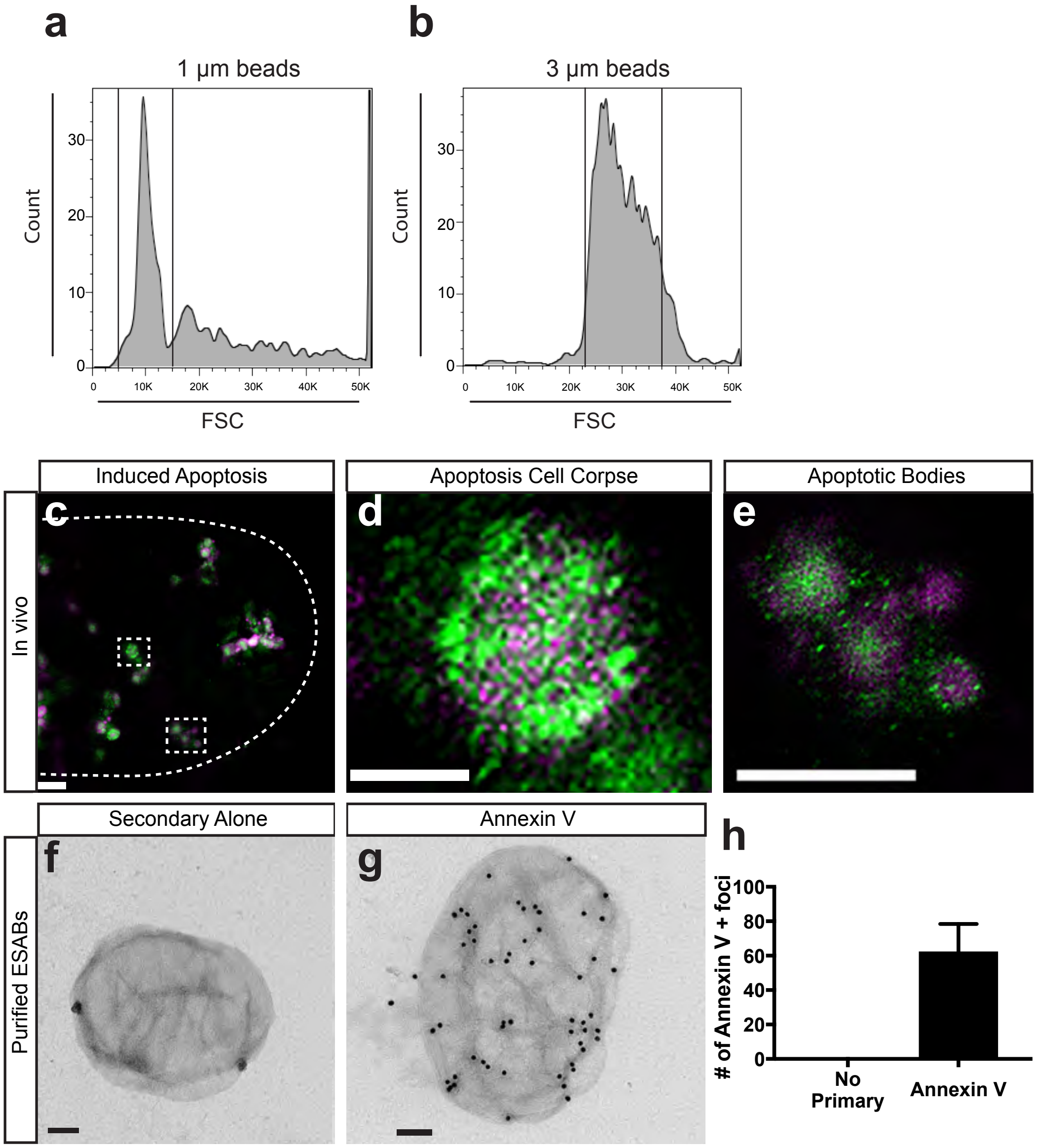

Supplementary Figure 6. Externalization of Phosphatidylserine on Apoptotic Stem Cells and ESABs (a-b) Histogram plots of 1.1μm and 3μm beads that define the gates used to describe the size of the purified ESABs in Figure 5j. (c-e) Maximum intensity projections of *Et(Gal4-VP16)zc1036a* (magenta) and Annexin V (green), (scale = 50μm in a, and 5μm in b-c). (f-g) Transmission electron micrographs of AnnexinV immunogold labeling on whole-mount purified apoptotic bodies (scale = 250nm). (h) Quantitation of Annexin V foci on the ESABs. Error bars represent SD.
