# Supplementary material for: Stem cell proliferation is induced by apoptotic bodies from dying cells during epithelial tissue maintenance
Source: Nat Commun. 2019 Mar 5;10:1044. doi: 10.1038/s41467-019-09010-6 (PMC6400930; doi:10.1038/s41467-019-09010-6)
Supplement: Supplementary file 3 — Description of Additional Supplementary Files [file 41467_2019_9010_MOESM3_ESM.pdf]

Description of Additional Supplementary Files

Supplementary Movie 1. Production of epithelial stem cell-derived apoptotic bodies in a living tissue. Maximum intensity confocal projection of time-lapse imaging of *Et(Gal4-VP16)*^{zc1036a}*;Tg(UAS-E1b:nsfB-mCherry)* after induced apoptosis. Images were acquired every 3 minutes for 2.5 hours.

Supplementary Movie 2. Engulfment of epithelial stem cell-derived apoptotic bodies. Maximum intensity confocal projection of time-lapse imaging of *Et(Gal4-VP16)*^{zc1036a}*;Tg(UAS-E1b:nsfB-mCherry; Tg(p63:EGFP)* after induced apoptosis. Images were acquired every 10 minutes for 2 hours.

Supplementary Movie 3. Proliferation of p63-positive epithelial stem cells after engulfment of apoptotic bodies. Maximum intensity confocal projection of time-lapse imaging of *Et(Gal4-VP16)*^{zc1036a}*;Tg(UAS-E1b:nsfB-mCherry)* in combination with *Tg(p63:EGFP)* after induced apoptosis and washout of MTZ. Images were acquired every 12 minutes for 9 hours.

Supplementary Movie 4. Generation of epithelial stem cell-derived apoptotic bodies containing Wnt8a. Maximum intensity confocal projection of time-lapse imaging of *Et(Gal4-VP16)*^{zc1036a}*;Tg(UAS-E1b:nsfB-mCherry); Tg(hsp70l:wnt8a-GFP)*^{w34} after overexpression of Wnt8a and induced apoptosis. Images were acquired every 2 minutes for 2.5 hours.
