# Supplementary material for: Stem cell proliferation is induced by apoptotic bodies from dying cells during epithelial tissue maintenance
Source: Nat Commun. 2019 Mar 5;10:1044. doi: 10.1038/s41467-019-09010-6 (PMC6400930; doi:10.1038/s41467-019-09010-6)
Supplement: Supplementary file 8 — Reporting Summary [file 41467_2019_9010_MOESM8_ESM.pdf]

nature research

Corresponding author(s): George T. Eisenhoffer, NCOMMS-18-03050B

Last updated by author(s): Jan 27, 2019

Reporting Summary

Nature Research wishes to improve the reproducibility of the work that we publish. This form provides structure for consistency and transparency in reporting. For further information on Nature Research policies, see Authors & Referees and the Editorial Policy Checklist.

Statistics

For all statistical analyses, confirm that the following items are present in the figure legend, table legend, main text, or Methods section.

n/a	Confirmed	
☐	☒	The exact sample size (n) for each experimental group/condition, given as a discrete number and unit of measurement
☐	☒	A statement on whether measurements were taken from distinct samples or whether the same sample was measured repeatedly
☐	☒	The statistical test(s) used AND whether they are one- or two-sided<br>*Only common tests should be described solely by name; describe more complex techniques in the Methods section.*
☒	☐	A description of all covariates tested
☐	☒	A description of any assumptions or corrections, such as tests of normality and adjustment for multiple comparisons
☐	☒	A full description of the statistical parameters including central tendency (e.g. means) or other basic estimates (e.g. regression coefficient) AND variation (e.g. standard deviation) or associated estimates of uncertainty (e.g. confidence intervals)
☐	☒	For null hypothesis testing, the test statistic (e.g. F, t, r) with confidence intervals, effect sizes, degrees of freedom and P value noted<br>*Give P values as exact values whenever suitable.*
☒	☐	For Bayesian analysis, information on the choice of priors and Markov chain Monte Carlo settings
☒	☐	For hierarchical and complex designs, identification of the appropriate level for tests and full reporting of outcomes
☒	☐	Estimates of effect sizes (e.g. Cohen's d, Pearson's r), indicating how they were calculated

Our web collection on statistics for biologists contains articles on many of the points above.

Software and code

Policy information about availability of computer code

Data collection	n/a
Data analysis	n/a

For manuscripts utilizing custom algorithms or software that are central to the research but not yet described in published literature, software must be made available to editors/reviewers. We strongly encourage code deposition in a community repository (e.g. GitHub). See the Nature Research guidelines for submitting code & software for further information.

Data

Policy information about availability of data

All manuscripts must include a data availability statement. This statement should provide the following information, where applicable:

- Accession codes, unique identifiers, or web links for publicly available datasets
- A list of figures that have associated raw data
- A description of any restrictions on data availability

The source data underlying Figs 1j-k, 2c,g, and h, 3i and l, 4g-h, 5d and n, and Supplementary Figs 1b and d, 2 d and g, 3 a, e and c, 4 f-h, 5 d and i, and 6h are provided as a Source Data file. All relevant data are available from the authors upon request.

Field-specific reporting

Please select the one below that is the best fit for your research. If you are not sure, read the appropriate sections before making your selection.

☒ Life sciences ☐ Behavioural & social sciences ☐ Ecological, evolutionary & environmental sciences

Life sciences study design

All studies must disclose on these points even when the disclosure is negative.

Sample size	Sample sizes were determined from 12 years of previous experience and review of the literature that variability of responses of the larvae to such treatments can be as high as 20-30%. These same analyses and published studies suggest that at least a 30% difference in effect from control versus experimental treatments needs to be detected to provide statistically meaningful results. Therefore, we determined a sample size of at least 16 animals per group, with 2 groups (wild-type and experimental) and at least three replicates per experiment to achieve significance.
Data exclusions	No data were excluded from the anlaysis
Replication	All attempts at replication were successful and experiments were performed at least three independent times.
Randomization	Samples were allocated into experimental groups based on treatment with pharmacological agents, or differences in genetic background.
Blinding	Investigators were blinded to group allocation during data collection.

Reporting for specific materials, systems and methods

We require information from authors about some types of materials, experimental systems and methods used in many studies. Here, indicate whether each material, system or method listed is relevant to your study. If you are not sure if a list item applies to your research, read the appropriate section before selecting a response.

Materials & experimental systems

n/a	Involved in the study
☐	☒ Antibodies
☒	☐ Eukaryotic cell lines
☒	☐ Palaeontology
☐	☒ Animals and other organisms
☒	☐ Human research participants
☒	☐ Clinical data

Methods

n/a	Involved in the study
☒	☐ ChIP-seq
☐	☒ Flow cytometry
☒	☐ MRI-based neuroimaging

Antibodies

Antibodies used	Activated Caspase-3, BD Biosciences (559565), 1:700; Bromodeoxyuridine (BrdU), Abcam (ab6326), 1:100; Phospho-Histone H3 (H3P), Abcam (5176), 1:500; Tp63, Abcam (111449), 1:500; Tp63, Genetex (GTX124660), 1:500; Wnt8a, ABGENT (AP21770c), 1:200; Phospho-Histone H2A.X (Ser319), Millipore (05-636), 1:200; Annexin V, Abcam (ab14196), 1:200; Nucview 488 Caspase 3 Substrate, Biotium (30029), 10 µM.
Validation	Antibodies were validated using a positive control (induced death, division or Wnt8a signaling) and a calibration curve was used to define the optimal concentration, which is reported in the methods section. We assessed cross-reactivities of structurally similar antigens and negative controls have also been tested.

Animals and other organisms

Policy information about studies involving animals; ARRIVE guidelines recommended for reporting animal research

Laboratory animals	Zebrafish, Danio rerio, 4-5 day post-fertilization larvae were used, sex cannot be determined at this age.
Wild animals	n/a
Field-collected samples	n/a
Ethics oversight	The zebrafish used in this study were handled in accordance with the guidelines of the University of Texas MD Anderson Cancer Center Institutional Animal Care and Use Committee.

Note that full information on the approval of the study protocol must also be provided in the manuscript.

Flow Cytometry

Plots

Confirm that:

☒ The axis labels state the marker and fluorochrome used (e.g. CD4-FITC).

☒ The axis scales are clearly visible. Include numbers along axes only for bottom left plot of group (a 'group' is an analysis of identical markers).

☒ All plots are contour plots with outliers or pseudocolor plots.

☒ A numerical value for number of cells or percentage (with statistics) is provided.

Methodology

Sample preparation	Larvae were incubated in Trypsin EDTA 0.25% for 30 minutes, dissociated with a pestle and subjected to a 10 minute centrifugation at 650 x g. The supernatant was transferred and centrifuged for 2 minutes at 14500 x g. The supernatant was once again transferred and centrifuged for 1 hour at 14500 x g. The pellet was washed twice and suspended in dPBS. The purified fraction containing the epithelial stem cell-derived (ESABs) apoptotic bodies was characterized using flow cytometry.
Instrument	Beckman Coulter Gallios
Software	FlowJo
Cell population abundance	The number and size distribution of mCherry positive ESABs was determined after purification and compared to extracellular vesicles isolated from zebrafish larvae under homeostatic conditions.
Gating strategy	Beads of known sizes 1.1 (Sigma LB11-1mL) and 3µm (Sigma LB30-1mL) were used as standards to set gates for determining the size of the apoptotic bodies, and a figure exemplifying this strategy has been added to Supplmentary Figure 6a-b.

☒ Tick this box to confirm that a figure exemplifying the gating strategy is provided in the Supplementary Information.
